# Supplementary material for: Colchicine Use and Major Adverse Cardiovascular Events in Male Patients with Gout and Established Coronary Artery Disease: A Veterans Affairs Nested Retrospective Cohort Study
Source: Gout Urate Cryst Depos Dis. Author manuscript; Available in PMC 2026 Aug 18. (PMC13480397; doi:10.3390/gucdd1010003)
Supplement: Supplementary file 1 [file NIHMS2203539-supplement-HoEtAl_Supplementary.pdf]

Table S1. Comparison of baseline characteristics between all Quartiles (1–4).

Variable	Quartile 1 (*n* = 60)	Quartile 2 (*n* = 60)	Quartile 3 (*n* = 60)	Quartile 4 (*n* = 59)
Age, years ± SD	79 ± 10	78 ± 9	78 ± 10	76 ± 9
Body Mass Index, kg/m^2 ± SD	30.8 ± 4.6	30.6 ± 6.6	31.2 ± 5.8	31.0 ± 5.1
Diabetes Mellitus	40.7%	44.1%	48.3%	39.0%
Hypertension	91.5%	88.1%	90.0%	84.7%
Systolic Blood Pressure, mmHg ± SD	131 ± 21	130 ± 28	132 ± 20	129 ± 20
Diastolic Blood Pressure, mmHg ± SD	74 ± 11	74 ± 15	75 ± 13	73 ± 12
Dyslipidemia	71.1%	83.1%	70.0%	81.4%
LDL Cholesterol, mg/dL ± SD	116 ± 46	94 ± 26	105 ± 38	94 ± 26
Prior Myocardial Infarction	41.7%	49.2%	44.1%	49.2%
Prior Coronary Artery Bypass Graft	26.7%	33.3%	28.3%	23.7%
Prior Percutaneous Coronary Intervention	28.3%	35.0%	38.3%	35.6%
Chronic Kidney Disease	41.4%	44.8%	41.7%	44.1%
Serum Creatinine, mg/dL ± SD	1.5 ± 0.8	1.5 ± 0.6	1.6 ± 0.6	1.4 ± 0.5
History of Tobacco Use	83.3%	78.0%	83.3%	81.4%
Current Tobacco Use	16.9%	15.3%	8.5%	18.6%
Allopurinol Use	36.7%	20.3%	33.3%	18.6%
Serum Uric Acid, mg/dL ± SD	8.2 ± 2.0	8.9 ± 2.0	8.7 ± 2.3	8.9 ± 2.0
NSAID Use	38.3%	30.0%	28.3%	23.7%
Aspirin Use	55.0%	63.3%	58.3%	62.7%
Statin Use	53.3%	65.0%	66.7%	74.6%
ACE inhibitor Use	38.3%	61.7%	53.3%	55.9%
β-blocker Use	70.0%	63.3%	78.3%	72.9%
Any Antihypertensive Use	55.0%	70.0%	86.7%	69.5%
Mean Daily Colchicine Dose, mg ± SD	0.73 ± 0.31	0.78 ± 0.26	0.66 ± 0.18	0.69 ± 0.22
Time on Colchicine, % of observation period ± SD	11.4 ± 4.9	31.3 ± 7.4	56.6 ± 7.8	88.5 ± 9.9

Continuous data are shown as mean ± standard deviation. Categorical data are shown as a percentage of the total subgroup. SD, standard deviation; LDL, low-density lipoprotein; NSAID, nonsteroidal anti-inflammatory drug, ACE, angiotensin-converting enzyme.

Table S2. Total Major Adverse Cardiovascular Events and Component Events in Colchicine Users and Nonusers.

Major Adverse Cardiovascular Event	Nonusers (*n* = 116)	Colchicine Users (*n* = 239)
Total First Major Adverse Cardiovascular Events	17	43
Myocardial Infarction	7	24
Coronary Artery Bypass Graft	3	9
Cerebrovascular Accident	6	6
All-cause mortality	2	8

The Total First Major Adverse Cardiovascular Events row displays the number of patients experiencing first-observed MACE within each group. Subsequent events for each individual patient were censored for logistic regression and Kaplan Meier analysis.

Table S3. Total Major Adverse Cardiovascular Events and Component Events in Each Colchicine-use Quartile.

Major Adverse Cardiovascular Event	Quartile 1 (*n* = 60)	Quartile 2 (*n* = 60)	Quartile 3 (*n* = 60)	Quartile 4 (*n* = 59)
Total First Major Adverse Cardiovascular Events	10	15	13	5
Myocardial Infarction	7	7	7	3
Coronary Artery Bypass Graft	2	3	3	1
Cerebrovascular Accident	0	2	3	1
All-cause mortality	2	5	1	0

The Total First Major Adverse Cardiovascular Events row displays the number of patients experiencing first-observed MACE within each quartile. Subsequent events for each individual patient were censored for logistic regression and Kaplan Meier analysis.
